# Prolactin levels and agressive behaviour in men with Schizophrenia

**DOI:** 10.1192/j.eurpsy.2023.946

**Published:** 2023-07-19

**Authors:** I. Bouguerra, E. Khelifa, A. Adouni, Y. Sellaouti, H. Abaza, H. Ben Ammar, L. Mnif

**Affiliations:** F pyshciatry departement, Razi Hospital, Mannouba, Tunisia

## Abstract

**Introduction:**

Recent studies find a high level of prolactin in naive patients with consequences on their behavior. These results have shed light on new etiopathogenic avenues in schizophrenia and suggested new preventive approaches.

**Objectives:**

The objective of our work was to investigate the links that may involve prolactin levels to agressive behavior in patients followed for antipsychotic-naïve schizophrenia or in therapeutic discontinuation.

**Methods:**

We conducted a one-year descriptive and cross-sectional study of thirty male patients hospitalized for a treatment-naïve psychotic relapse or who had been discontinued for more than two months. These patients were assessed using a questionnaire as well as the Overt Agression Scale (OAS). A blood sample was taken to specify the prolactin level.

**Results:**

Eleven patients were aggressive (37%). Seven patients (23%) had hyperprolactinemia. Hyperprolactinemia was also inversely associated with aggression since inversely significant correlations were objectified for prolactinemia and respectively the OAS score and the verbal aggression subscore (Rho=-0.391 ; p=0.033) and (p=0.016, Rho=-0.438). The score of aggressiveness towards others also evolved inversely to the prolactin level with a p close to significance (p =0.056).

**Conclusions:**

Our results support the hypothesis of a probable action of prolactin as a protective factor against aggression. High prolactin levels may therefore represent a diagnostic lead for a particular profile of a certain patient group with a particular course. However, this subject is still unresolved in the literature and future studies seem necessary.

**Disclosure of Interest:**

None Declared

